# Molecular characterization of influenza virus circulating in Nepal in the year 2019

**DOI:** 10.1038/s41598-024-58676-6

**Published:** 2024-05-07

**Authors:** Rachana Mehta, Bimalesh Kumar Jha, Balkrishna Awal, Ranjit Sah, Lilee Shrestha, Chhoting Sherpa, Smriti Shrestha, Runa Jha

**Affiliations:** National Public Health Laboratory Teku, Kathmandu, Nepal

**Keywords:** Influenza, RT PCR, Strain, Lineage and pandemic, Microbiology, Molecular biology, Medical research

## Abstract

Influenza (sometimes referred to as “flu”) is a contagious viral infection of the airways in the lungs that affects a significant portion of the world's population. Clinical symptoms of influenza virus infections can range widely, from severe pneumonia to moderate or even asymptomatic sickness. If left untreated, influenza can have more severe effects on the heart, brain, and lungs than on the respiratory tract and can necessitate hospitalization. This study was aimed to investigate and characterize all types of influenza cases prevailing in Nepal and to analyze seasonal occurrence of Influenza in Nepal in the year 2019. A cross sectional, retrospective and descriptive study was carried out at National Influenza Center (NIC), National Public Health Laboratory Kathmandu Nepal for the period of one year (Jan–Dec 2019). A total of 3606 throat swab samples from various age groups and sexes were processed at the NIC. The specimens were primarily stored at 4 °C and processed using ABI 7500 RT PCR system for the identification of Influenza virus types and subtypes. Data accessed for research purpose were retrieved from National Influenza Centre (NIC) on 1st Jan 2020. Of the total 3606 patients suspected of having influenza infection, influenza viruses were isolated from 1213 (33.6%) patients with male predominance. The highest number of infection was caused by Influenza A/Pdm09 strain 739 (60.9%) followed by Influenza B 304 (25.1%) and Influenza A/H3 169 (13.9%) and most remarkable finding of this study was the detection of H5N1 in human which is the first ever case of such infection in human from Nepal. Similar to other tropical nations, influenza viruses were detected year-round in various geographical locations of Nepal. The influenza virus type and subtypes that were in circulation in Nepal were comparable to vaccine candidate viruses, which the currently available influenza vaccine may prevent.

## Introduction

Influenza is an acute viral disease of the respiratory tract which is caused by influenza virus^1^. There are four different serotypes of this virus, which belongs to the Orthomyxoviridae family: influenza A, influenza B, influenza C, and influenza D. Although the influenza A and B viruses are the ones that cause the common seasonal flu outbreaks in people, influenza C infections often cause minor symptoms and are not linked to widespread human flu epidemics. Influenza D viruses affect cattle largely and are not known to be able to infect or sicken humans^2^. Based on the antigenic characteristics of the surface glycoproteins hemagglutinin (HA) and neuraminidase (NA), influenza viruses of type A can be further divided into two major groups, namely low pathogenic seasonal influenza (A/H1N1, A/H1N1 pdm09, A/H3N2) and highly pathogenic influenza virus (H5N1, H5N6, H7N9)^3,4^. Eleven NA subtypes (N1-N11) and 18 HA subtypes (H1-H18) have so far been identified^5^.

Seasonal epidemics are generally caused by influenza A viruses, including subtypes H1N1pdm09 and H3N2, and influenza B viruses, specifically lineages B-Yamagata and B-victoria^6^. According to current estimates, seasonal influenza kills between 250,000 and fifty thousand individuals worldwide each year, affecting between 10 and 20% percent of the global population^7,8^. The influenza A virus undergoes a minor antigenic change, namely antigenic drift from year to year and may also undergo a major changes, termed an antigenic shift as occurred with the emergence of the swine origin A/H1N1 pdm 09 influenza virus, which was considered a major pandemic threat to human health^9^. Influenza related complications including hospitalization and deaths are often seen in very young, elderly and people with underlying medical conditions^10^.

Similar to the tropical and sub-tropical regions of Southeast Asia, Nepal experiences year-round influenza B, A/H3N2, and A/H1N1 pdm09 circulation, with a peak from July to November. However, the rate of infection transmission reach peak during the post-rain and winter season of Nepal^8^.

As each influenza season is characterized by specific patterns of circulating influenza viruses, the identification and characterization of influenza viruses is essential in order to develop effective vaccines against the influenza strains predicted to circulate in the upcoming season^6^. Therefore, this study is aimed to investigate influenza cases, to characterize influenza types and subtypes and also to analyze the seasonal occurrence of influenza infection in Nepal.

## Methods and methodology

### Study design and sample collection

A retrospective, descriptive and cross sectional study was carried out at the National Influenza Center (NIC), National Public Health Laboratory (NPHL), Kathmandu, Nepal for the period of one year (January–December 2019). The data were obtained from the National Influenza centre for the research purpose on 1st Jan 2020. A total of 3606 throat swab specimens were collected from sentinel site of National Influenza Surveillance Network and NPHL. The samples obtained at sentinel sites were transported to NPHL in a cold chain box and preserved in viral transport media within 24 h where they were kept at 4 °C until processing. According to the WHO case criteria for ILI and SARI, patients included in the study either had pneumonia or influenza-like illness (ILI), which includes fever (> 38 °C), cough, running nose, chills, and sore throat within last seven days^11^.

### Sample processing and virus identification

All samples in viral transport media were aliquoted into two micro centrifuge tubes. One was kept at − 70 °C while the other was utilized for RNA extraction. Using the QIAamp® Viral RNA Mini Kit (QIAGEN GmbH, Hilden, Germany) and following the manufacturer's instructions, influenza viral RNA was extracted. Applied Bio systems TM 7500 Real-Time PCR System and AgPath-IDTM One-step RT-PCR Kit from Thermo Fisher Scientific, USA, were used to identify the influenza virus. First, influenza A and B viruses were screened for in the retrieved RNA. Further classification into subtypes and lineages was done on the sample that tested positive for influenza A/B. Influenza A positive sample were further tested for Pdm A, Pdm H1, A/H3 while samples exhibiting influenza B positivity were checked for B/Yamagata and B/Victoria lineage. Due to limited supply of PCR kit, only 83 influenza B positive samples were further processed to determine their lineages. The primers and probes used in the reaction mixture for identification of various influenza virus types, subtypes and lineages (H1N1, H3N2, H1N1pdm09, Influenza B, B/Victoria, B/Yamagata) were provided by US Center for Disease Control and Prevention (CDC) through the IRR and the assays were carried out according to manufacturer’s protocols. Cycle threshold (Ct) values greater than 40 were regarded as positive in samples^12^.

In accordance with established guidelines and regulations, all methods employed in this study were conducted after obtaining approval from Nepal Health research council. The research procedures followed ethical standards and adhered to the recommended protocols outline in Helsinki declaration.

### Ethical consideration

This study is a Laboratory based retrospective study conducted at National Public Health Laboratory. Ethical approval was obtained from Ethical Review Committee, Nepal Health Research Council (NHRC ref. no. 1966 on 18 March 2020) and no individual consent from participants were taken as data was extracted from ongoing Global Influenza Surveillance Program with the approval of Director of the National Public Health Laboratory. All the samples were anonymized and only code number generated in Global Influenza Surveillance Program (Nepal) were used for analysis.

Therefore, written informed consent is deemed unnecessary for this research as this study was approved by both NHRC and NPHL.

## Result

### Demographic characteristics of patients

During the year 2019, a total of 3606 throat swab specimens from 1880 males and 1726 females were tested at National Public Health Laboratory for Influenza virus infection, among which 1213 samples tested positive (33.6%). Gender distribution and clinical findings (SARI or ILI) of samples received and positive cases is seen in Table 1.

**Table 1:** Gender wise participants of Case.

	Gender	Number of sample	Positivity (%)	Total
SARI	Male	1418	51.8%	2736
	Female	1318	48.2%	
ILI	Male	462	53.1%	870
	Female	408	46.9%	
Total		3606	100%	3606

Age-wise distribution of influenza suspected cases as well as influenza positive was observed higher in age group 15–45 years (Table 2).

**Table 2:** Age-wise distribution of influenza positive cases.

Age	Number of Sample	Positivity %
> 45	483/1454	33.2
15–45	601/1535	39.2
5–14	79/311	25.4
0–4	50/306	16.1

**Figure 1:**
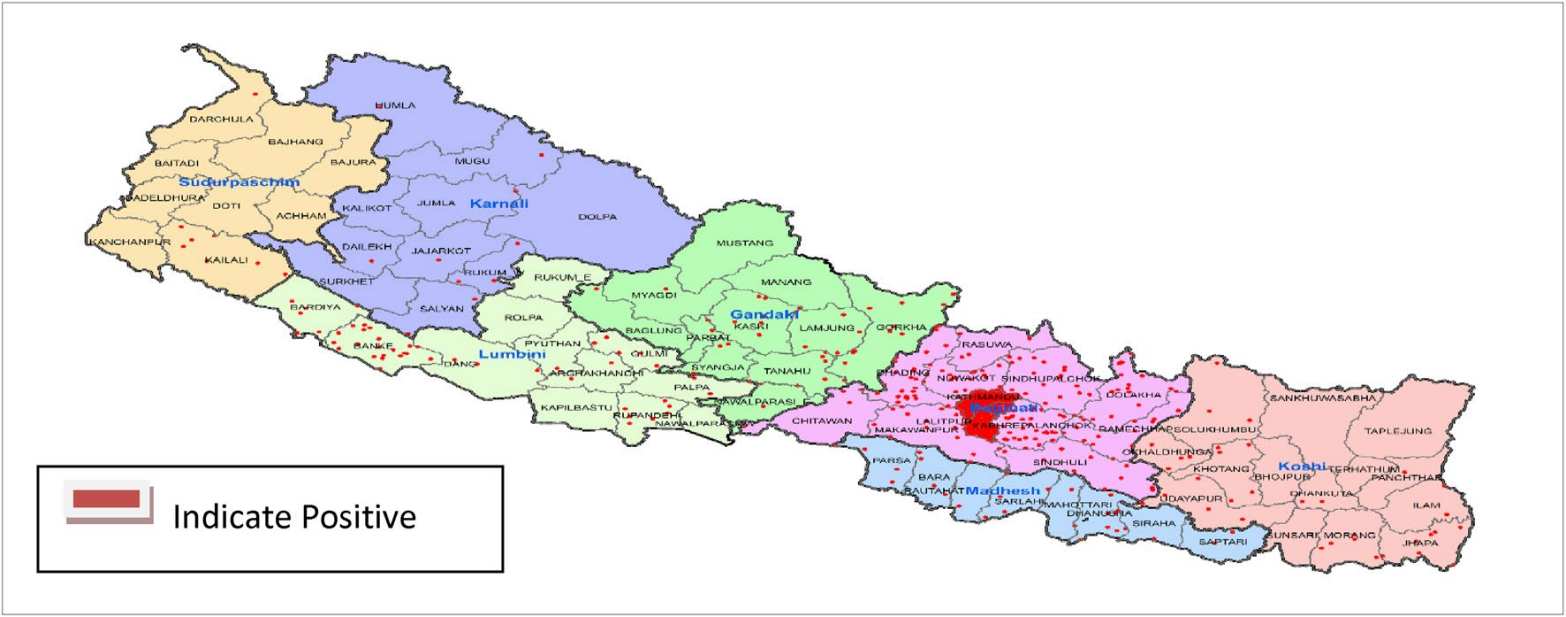
District wise transmission of influenza viruses, 2019 (Map was created independently using ArcGIS Version 10.5, arcgis.com).

National Public Health Laboratory received samples from 73 districts in 2019. However, positive cases for influenza infection were confirmed from 56 districts only. District wise distribution of positive cases is shown in Fig. 1.

### Prevalence of influenza virus

Among 1213 influenza positive cases, Influenza type A accounted for 909 (74.9%) cases of infection whereas influenza type B was found in 304 (25.1%) cases. Among Influenza type A, most positive cases 739 (60.9%) were found infected with influenza A/Pdm09 subtype whereas 169 (13.9%) were positive for influenza A/H3 subtype. Among the 304 cases of influenza B infection, only 83 isolates were further differentiated into lineage and this revealed higher number of viruses from B/Victoria lineage 60 (4.95%) in comparison to B/Yamagata lineage 23 (1.89%) (Table 3).

**Table 3:** Strain and lineage of the influenza virus.

Influenza viruses	No. of positive cases		Percentage (%)	
Influenza A/Pdm09	739	909	60.9%	74.9%
Influenza A/H3	169		13.9%	
H5N1	1		0.08%	
Influenza B virus	221	304	18.22%	25.1%
Inf B/Vic	60		4.95%	
Inf B/Y	23		1.89%	
Total	1213		100	

### Seasonal distribution of influenza viruses

**Figure 2:**
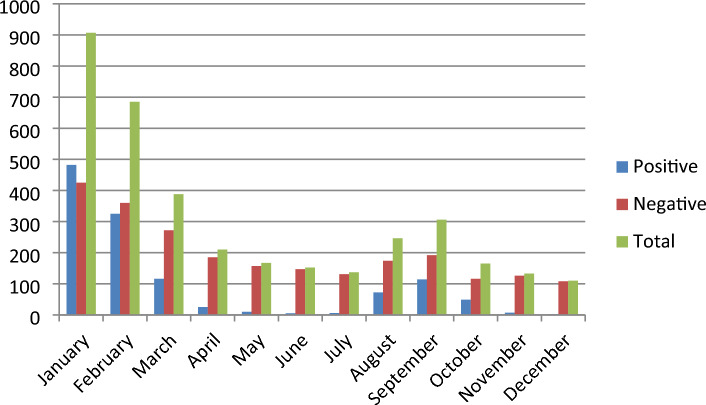
Month wise analysis of influenza cases in year 2019 (N = 3606).

Figure 2, illustrates the distribution of cases of influenza in different months throughout the year 2019. It can be observed that higher numbers of influenza cases were reported with two peaks in the year 2019, first during the month of January–February and second during the month of August–September. The highest numbers of cases were recorded in the month of January and February. Positivity rate was 53.1% in January, 47.4% in February and 37.3% in the month of September. The least number of influenza positive cases were reported in the months of May, June, July, November and December.

**Figure 3:**
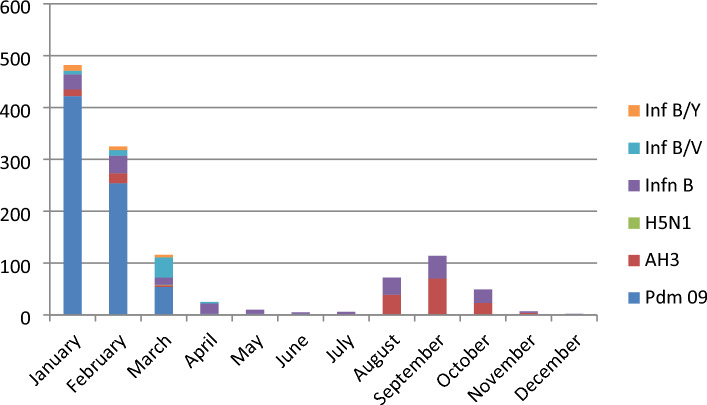
Influenza subtypes identified in different moths of the year 2019 (n = 1213).

Influenza A/Pdm09 strain was found predominant during the first peak whereas InfA/H3 was predominant during second peak. (Fig. 3) A single case of H5N1 was also identified which was first human case of H5N1 reported from Nepal.

## Discussion

In Nepal, influenza is one of the major causes of public health problems, yet little is known about its epidemiology. Following an outbreak, the influenza virus has been seen to re-assort with various types of genetic alterations, creating a new strain that may further fuel an epidemic^13^. According to the World Health Organization, the H1N1pdm09 or H3N2 subtype of the influenza A virus has been co-circulating in the European region throughout the 2018–2019 flu season^14^. However, either influenza A(H1N1) pdm09 or A(H3N2) dominated (2014/15–2016/17) of influenza season^15^. Every season, a new strain or genetic variation of the influenza virus reemerges, bringing with it novel antigenic features that may reduce vaccine-induced protection due to strain mismatches between the vaccine and circulating strains. To provide the best protection against influenza infection, it is required to identify and define the novel strain of virus and adjust the vaccine in accordance with the dominant strain. The Early warning response system (EWARS) in Nepal, established by the Epidemiology and Diseases control division (EDCD) in 1997 is a hospital based syndromic surveillance that received weekly reporting of number of cases and deaths of six priority diseases / syndromes including SARI. At present 118 sites are reporting to EWARS. In 2019, 10542 SARI were reported to EWARS from these sites^16^.

During the study period, influenza A/Pdm09 was the most predominant strain (53.1%) circulating in Nepal. A study conducted by Adhikari et.al also reported similar finding according to which pandemic influenza AH1N1 dominated in the year 2009 in Nepal^17^. However, different from previous study by Upadhayay et al. and Jha et al. which showed influenza A/H3 was responsible for 60.1% and 51.0% of the total infection in year 2014 and 2016 in Nepal respectively^8,15^. According to a study from tropical Asia, influenza A predominated over influenza B between 2007 and 2013, which is consistent with our study^18^. In this investigation, we found that the B/Victoria lineage of the influenza type B virus predominated over the B/Yamagata lineage, which was different from the study carried out by Jha et.al in the year 2016 in Nepal^15^. Although our research reveals that B/Victoria lineage predominated over B/Yamagata which is similar to study conducted by Northern Hemisphere during the 2016–2017 season^19^. The prevalence rates given in different studies may differ since they were conducted at diverse times and locations.

This study illustrates circulations of influenza infection throughout the year with the first peak in January–February followed by August–September. The study conducted by Jha et al. in the past from the same institution also demonstrated that Nepal observes two peaks of influenza infection round the year; first in the month of January and second in the month of July–August^15^. In our study second peak was observed in the month of August–September rather than July–August which is slightly different from previous study. Similar to our findings, a study from mainland China by Sun et al. showed two influenza activity peaks, one in the winter and the other in the spring of each monitoring year^20^. In contrast, the temperate region of the Japanese mainland only suffers a single surge in influenza activity during the winter months^21^. Numerous research looked at the seasonal patterns of influenza, but the mechanisms by which new virus strains arise and propagate are still not fully known but likely include a combination of climatic conditions, susceptibility of the population and virus characteristics^22,23^. From the perspective of public health, knowledge of the seasonality of pathogens is essential to help determine the timing of interventions, especially in a nation with a variety of climatic and economic conditions^24^. In this perspective, for optimizing influenza management tactics and for understanding the epidemiology and seasonality of influenza, a good influenza monitoring and surveillance system is crucial^25^.

One of the most remarkable findings of this study was the detection of H5N1 from human, which was the first ever case of such infection in human from Nepal. NPHL confirmed the Influenza A infection from throat swab sample of 21-year-old male patient admitted in Hospital. However, the patient could not be saved and died following respiratory complications on 29th March 2019. The subtype of the virus was tested negative for Influenza A/Pdm09 and Influenza A/H3, which was later confirmed at NIC of Japan, on 30 April, 2019 a WHO Collaborating Center for Influenza to be Influenza A/H5N1^26,27^.

Due to several restrictions on this work, including a lack of funding and resources, we were unable to carry out comprehensive genomic characterizations. However, to comprehend the epidemiology and seasonality of influenza and to optimize control methods, effective and ongoing influenza monitoring and surveillance systems are required.

## Conclusion and recommendations

In Nepal, influenza viruses have been found all year long in various regions, just like in other tropical countries (Supplementary Information). All forms of influenza viruses are in circulation, with the peak season occurring between January and March. To connect viral genetic changes with antigenic changes, it is required to compare the genetic patterns of the influenza virus throughout time.

### Supplementary Information

Supplementary Information.

## Data Availability

All necessary data are included in paper. The Datasets analyzed during the Current study are available in Global Influenza Surveillance Network. Remaining data will be provided by corresponding authors on reasonable request.
